# Tannin extracts from immature fruits of *Terminalia chebula Fructus *Retz. promote cutaneous wound healing in rats

**DOI:** 10.1186/1472-6882-11-86

**Published:** 2011-10-07

**Authors:** Kun Li, Yunpeng Diao, Houli Zhang, Shouyu Wang, Zhen Zhang, Bo Yu, Shanshan Huang, Hong Yang

**Affiliations:** 1 College of Life Sciences, Liaoning Normal University, Dalian 116029, PR China; 2 College of Pharmacy, Dalian Medical University, Dalian 116044, PR China

## Abstract

**Background:**

Tannins extracted from immature fruits of *Terminalia chebula Fructus Retz*. are considered as effective components promoting the process of wound healing. The objective of this study is to explore the optimal extraction and purification technology (OEPT) of tannins, while studying the use of this drug in the treatment of a cutaneous wound of rat as well as its antibacterial effects.

**Methods:**

The content of tannin extracts was measured by the casein method, and antibacterial ability was studied by the micro-dilution method in vitro. In wound healing experiment, animals in group Ⅰ, Ⅱ and Ⅲ were treated with vaseline ointment, tannin extracts (tannin content: 81%) and erythromycin ointment, respectively (5 mg of ointment were applied on each wound). To evaluate the process of wound healing, selected pharmacological and biochemical parameters were applied.

**Results:**

After optimal extraction and purification, content of tannin extracts was increased to 81%. Tannin extracts showed the inhibition of *Staphylococcus aureus *and *Klebsiella Pneumonia *in vitro. After excision of wounds, on days 7 and 10, the percent of wound contraction of group Ⅱ was higher than that of group Ⅰ. After being hurt with wounds, on days 3, 7, and 10, the wound healing quality of group Ⅱ was found to be better than that of group Ⅰ in terms of granulation formation and collagen organization. After wound creation, on day 3, the vascular endothelial growth factor expression of group Ⅱ was higher than that of group Ⅰ.

**Conclusion:**

The results suggest that tannin extracts from dried immature fruits of *Terminalia chebula Fructus Retz*. can promote cutaneous wound healing in rats, probably resulting from a powerful anti-bacterial and angiogenic activity of the extracts.

## Background

Wound healing may be a challenging medical issue that requires specialized treatment and care. Although tannin extracts have been used in improving the process of wound healing [1], the chemical components of the extracts and their mechanisms in vivo have not been completely understood till date. In recent years, for human and veterinarian use, many research studies have indicated an interest in exploring drugs obtained from plants with a high content of tannins. These are potentially useful in promoting the healing of wounds and burns. Tannins could promote cicatrisation of wounds through several cellular mechanisms: i) chelation of free radicals and reactive species of oxygen, ii) promoting contraction of the wound, and iii) increasing formation of capillary vessels and fibroblasts [2]. The immature fruit of *Terminalia chebula Fructus Retz *is found in Yunnan, Tibet, Guangdong, and Guangxi provinces of China and has been termed as Xiqingguo in Chinese. It is also found in Malaysia, Thailand, India, and Pakistan [3-6]. Its use for medicinal purposes has been historically accredited in Ayurvedic literature. In Thai folk, it is considered as a natural remedy for skin diseases, wound healing, and rejuvenation [7].

Many local factors can affect the development of wounds. Bacterial infection is the most important factor that can impact the development of wounds [8,9]. Recent researches suggest that bacteria release proteolytic enzymes to digest the connective tissue of skin, resulting in tissue necrosis and wound expansion. Tissue necrosis and liquefaction can promote bacterial growth. The above factors can interact with each other, aggravating the pathological condition and eventually resulting in extensive infection and sepsis. Infection usually involves bacteria with strong pathogenicity, such as *Staphylococcus aureus *and *Pseudomonas aeruginosa*, etc. On the other hand, *Escherichia coli *and *Klebsiella pneumoniae *are prone to produce extended-spectrum β-lactamases (ESBLs) [10]. It has been found that some plant phenolics, including flavonoids and tannins have antibacterial effects [11-13].

Cutaneous wound healing is a complex process, which consists of progression of inflammation, angiogenesis, collagen deposition, reepithlization, and tissue remolding [14]. The purpose of repairing events is to resist pathogens invasion, establish integrity of damaged tissue, and reconstruct physiological function of the skin [15].

Vascularization is a process that involves vascular endothelial cells differentiation and proliferation to form a new vascular system. The process lays an important foundation for wound healing. In human tissues, new vessels would stop growing after fulfilling the normal physiological needs. With a molecular weight of 34-45 KD, endothelial cell growth factor (VEGF) was considered as one of the important regulatory factors. VEGF can activate biological activities by forming dimmers with glycoprotein monomers undergoing disulfide bonds. VEGF is considered the strongest mitogen for the proliferation of vascular endothelial cells [16]. In recent times, several other factors, including the placenta growth factor (PIGF), VEGF-A, -B, -C, -D and -E were found to have similar functions. All of the factors were defined in terms of VEGF family [17]. The amino acid sequence is also considered as highly conserved. The factors bind homotyrosine kinase receptor, playing key roles in physiological and pathological processes of embryonic development and wound healing.

In our present work, we study an optimal extraction and purification of tannin extracts from the immature fruit of *Terminalia chebula Fructus Retz*. To study the efficiency of tannin extracts on wound healing, angiogenesis, and VEGFA expression, we used rat model of wound excision. We also analyzed antibacterial effects of tannin extracts.

## Methods

### Plant material

In July, 2010, immature fruits of *Terminalia chebula Fructus Retz*. were purchased from the Dalian Nepstar Chain Drug Store of Liaoning province in China. The fruits were identified by Dr. Yun-Peng Diao, a professor of Dalian Medical University. The voucher specimen was deposited in a pharmacognosy laboratory along with a given specimen number XT001.

### Optimization of extraction and purification technology and preparation of extracts

Tannin extracts were temperature-sensitive. According to requirements of production, temperature during extraction was set to 50°C, and water was considered as the extraction solvent. Four factors can affect extraction:(A) duration of extraction, (B) maceration time, (C) extract-solvent ratio, and (D) number of extraction. The study was conducted in accordance with the orthogonal test of four factors at three different levels. The immature fruit powder of *Terminalia chebula Fructus Retz*. (10 g in weight) was extracted with water (100 ml) at 50°C. Thereafter, the extracts were weighted. Content of tannin extracts was measured and optimal extraction and purification technology (OEPT) was determined. The extracts were added to ethanol of 95% concentration, and the concentration of extract solutions were diluted to 80%. The extract solution was deposited for 12 hours and centrifuged at 4000 rpm for 10 min. After filtration, the content of tannin extracts was analyzed by the casein method.

Table 1 enlists the data of orthogonal test.

**Table 1:** The orthogonal test of four factors at three different levels

Factors	A duration of extraction (hour)	B maceration time(min)	C extract-solvent ratio(times)	D number of extraction(times)
1	1	20	10	1
2	1.5	40	15	2
3	2	60	20	3

### Determination of content of tannin extracts

The content of tannin extracts was measured by the casein method described in China Pharmacopoeia [18].

### Preparation of reference solution

In a 25 ml brown measuring flask, reference substance solutions (0.05 g gallic acid per ml) in aliquots of 1.0 ml, 2.0 ml, 3.0 ml, 4.0 ml, and 5.0 ml were separately placed. Then, 1 ml phosphotungstomolybdic acid was added to each of these aliquots. Thereafter, 11 ml, 10 ml, 9 ml, 8 ml, and 7 ml of water were respectively added to each of these aliquots. Finally, they were diluted to a volume of 25 ml using 29% Na_2_CO_3 _solution. Absorbance of the reaction mixture was read at 760 nm. A calibration curve of gallic acid (ranging from 1 to 10 μg/ml) was prepared.

### Procedure of determination

Total phenol content was determined as follows: 2 ml sample solution was poured into a 25 ml brown measuring flask. Then, 10 ml water was added to it and absorbance was measured. The content of the mixture was determined using a standard curve. Non-adsorbed polyphenol content was determined as follows: 25 ml sample solution was poured into a 100 ml stoppered conical flask, containing previously added 0.6 g casein. The mixture was stored in a water bath at 30°C for 1 hour; 2 ml filtrate was accurately measured in a 25 ml brown measuring flask and 10 ml of water was added. After measuring the absorbance, the content of the mixture was determined using a standard curve. Tannin content of the solution was calculated with the following formula: Content of the tannin extracts = (Total phenol content)-(Non-adsorbed polyphenol content).

### Antibacterial activity

Antibacterial activity of the tannin extracts was studied after taking into account *staphylococcus aureus *(ATCC25923) and *Klebsiella pneumonia *(ATCC700603). Antibacterial ability was studied by the micro-dilution method. Using reference antimicrobial drugs like penicillin and cefoperazone sodium (NCCLS, 2000), minimum inhibitory concentration (MIC) and minimum bactericidal concentration (MBC) were determined. After activation and culture development for 24 hours, 2% of *Staphylococcus aureus *and *Klebsiella Pneumonia *were separately inoculated in broth-based media containing minimum inhibitory concentration of tannins extracts at 37°C with shaking at 120 rpm. After adding tannin extracts and centrifugation, a total of 3 ml medium was collected at intervals of 4 h, 24 h, and 30 h. The collected bacteria sediment was washed with phosphate buffer three times. The obtained bacteria were used for preparing specimen. Morphologic and structure changes of the bacteria were observed, using a KYKY-1000B scanning electron microscope.

### Animal preparation and treatment

Adult male Sprague-Dawley rats, weighing 200-220 g, were supplied by the Animal Experimental Center of Dalian Medical University. Before conducting the study, each rat was housed in an individual cage in the same room for 1 week. The controlled environment included the following parameters: 12-hour light/dark cycle, 23 ± 2°C, and relative humidity 70%. The rat was given free access to a standard laboratory diet and water. All experimental procedures were approved by the Animal Research Ethics Committee of Dalian Medical University, Dalian, China (DMU10/02/23).

The rat was anesthetized with intra-peritoneal injection of 10% chloral hydrate (0.3 ml/100 g). The dorsal surface of the rat was shaved, and the underlying skin was cleaned with povidone iodine. An acute excision circle of 1.5 cm in diameter was engraved on the wound, using a scalpel blade on the back of the rat. The rats were then randomly divided into group I, II, and III (36 rats in each group). The wound of group I was treated with vaseline ointment at a dosage of 5 mg per wound, serving as a negative control. The wound of group II was treated with tannin extracts at a dosage of 5 mg per wound. The wound of group III was treated with erythromycin ointment (Approval Number: H11021246, Beijing Shuangji Pharmacy Co,. Ltd, China) at a dosage of 5 mg per wound, serving as a positive control. All drugs were applied topically every other day, until a complete healing of wounds was achieved.

### Wound measurement

After wound creation, six rats of each group were randomly selected and sacrificed on days 1, 3, 7, 10, 14, and 21, respectively. The wound diameter was measured, and the area (cm^2^) within the boundary was calculated planimetrically. The percentage of wound contraction was determined using the following formula: percentage of wound contraction = [(original wound area - unhealed area)/original wound area] × 100%.

### Histological examination of excised tissue

The excised wound tissue was fixed in 10% neutral buffered formalin. It was dehydrated in graded ethanol, cleared in xylene, and embedded in paraffin. On the glass slides, five-micron-thick sections of the epidermis, dermis, and subcutaneous panniculus carnosus muscle have been mounted. After dewaxing the sample, it was rehydrated to distilled water and stained with hematoxylin and eosin. All subsequent analyses were performed by an experienced pathologist without knowledge of the previous treatments. Based on the degree of re-epithelization, granulation tissue formation, and collagen organization, a five-tiered grading system was adopted to evaluate the historical differences of different samples, as showed in Table 2[19,20].

**Table 2:** Score of historical evaluation

Score	Re-epithelialization	Granulation tissue formation	Collagen organization
0	None	None	None
1	Migrating	Hypo cellular with few vessels	Trace
2	Partial stratum corneum	Many vessels and some cells	Slight
3	Hypertrohic	Many fibroblasts, some fibers	Moderate
4	Complete and normal	More fibers, few cells	Marked

### Immunohistochemical analysis of VEGFA expression

The selected wound tissue sections were deparaffinized and redehydrated after holding at 60 °C for 2 h. Thereafter, 3% H_2_O_2 _in methanol was used for 10 min to prevent endogenous peroxidase activity. The section was boiled in 0.01 mol/L citric acid for 20 min for retrieving antigen. To prevent nonspecific binding, normal goat serum was applied at 37°C for 10 min. The section was then reacted mouse anti-rat VEGFA monoclonal antibody (diluted 1:5, Abcam, UK) at 37°C for 1 h. After washing with phosphate buffered saline, the section was first incubated with bio-tinylated goat anti-mouse antibody (Beijing Zhongshan Biology Technology Co., Ltd, China) at 37°C for 30 min. Then, the same process was repeated using horseradish peroxidase-labeled streptavidin at 37°C for 30 min. After staining with 3, 3'-diaminobenzidine (DAB)/H_2_O_2 _and hematoxylin, the section was dehydrated, cleared, and mounted for viewing. For VEGFA analysis, the section was first examined under a microscope (100 × magnification) to identify the highest positive expression in the wound. Then, five areas of the highest expression were selected for evaluating under a microscope (400 × magnification). To calculate the area, density mean, and integrated optical density of positive expression, image was analyzed using Image-pro-plus 6.0 software (Media Cybernetics, USA). The average result of the five areas was recorded in terms of the statistic data of this wound tissue.

### Analysis of VEGFA mRNA expression by RT-PCR

cDNA was synthesized using RT-PCR kit (TaKaRa, Japan) according to the manufacturer's protocol. Total RNA was isolated from homogenized excised tissues using Trizol (BBI, USA). The oligonucleotide sequences of the primers of VEGFA were 5'-TGCACCCACGACAGAAGGGGA-3' for sense and 5'- TCACCGCCTTGGCTTGTCACAT-3' for antisense. On the other hand, used as a control, the sequences of primer of GAPDH were 5'-GGCCGTGAAGTCGTCAGAAC-3' for sense and 5'-GCCACGATGCCCAGGAA-3' for antisense. PCR conditions were expressed as follows: denaturation at 95°C for 3 minutes, and then 30 cycles of denaturation for 20 seconds at 94°C, annealing for 30 seconds at 55°C, and extension for 30 seconds at 72°C. Five μl PCR products were separated by electrophoresis using 1.0% agarose gel and photographed under ultraviolet radiation light. Band intensity was measured by using Gel-Pro Analyzer 6.0 software (Media Cybernetics, USA) and was normalized to those for GAPDH.

## Results

### Preparation of extracts and determination of optimal extraction and purification

According to an orthogonal test, optimal extraction and purification was determined at three different levels using four factors. Table 3 represents the results of the orthogonal test. The results showed that the order of influencing factors as following: D > B > C > A. Number of extraction was more important than other factors for extraction, and duration of extraction was least important. Therefore, an extraction lasting for duration of 1 hour could be considered as appropriate for the test. The OEPT was set (A_1_B_1_C_2_D_3_) as following: the immature fruit powder (10 g) was macerated for 20 min and then extracted three times with water (extract-solvent ratio = 1:15) for a time duration of 1 h at 50°C. Ethanol of 80% concentration was added to these extracts and placed for 12 h. The extracted solution was centrifuged at 4000 rpm for 10 min and filtered. The content of tannin extracts was analyzed by casein method.

**Table 3:** The result of orthogonal test

No	A	B	C	D	extract weight(%)y1	TTC(%)y2	score z
1	1	1	1	1	56.76	22.54	73.79
2	1	2	2	2	69.10	24.97	85.14
3	1	3	3	3	73.18	30.92	98.65
4	2	1	2	3	72.14	31.63	99.44
5	2	2	3	1	34.94	26.04	68.46
6	2	3	1	2	64.81	24.73	82.34
7	3	1	3	2	62.08	30.09	91.09
8	3	2	1	3	65.58	28.54	89.99
9	3	3	2	1	52.24	24.25	74.56
K1	257.58	264.32	246.12	216.81			
K2	250.24	243.59	259.14	258.57			
K3	255.64	255.55	258.20	288.08			
k1	85.86	88.11	82.04	72.27			
k2	83.41	81.20	86.38	86.19			
k3	85.21	85.18	86.07	96.03			
R	2.45	6.91	4,34	23.76			

Z = (y1/y1max) × 40 + (y2/y2max) × 60

### Determination of content of tannin extracts

**Figure 1:**
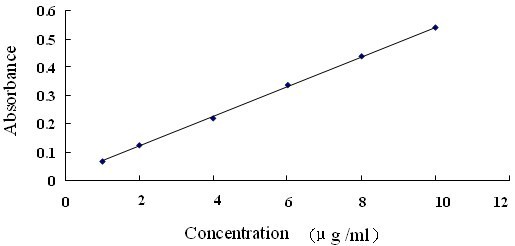
**The calibration curve of gallic acid**. linear range (1~10 μg/ml), Regression equation(Y = aX + b): y = 52.496x + 0.0163, R^2 ^= 0.9993(n = 6)

Regression equation was calculated in the form Y = aX + b. In the formula, X and Y are concentration of standard solution (mg/mL) and corresponding absorbance. a and b correspond to slope and intercept, respectively. A calibration curve of gallic acid (ranging from 1 to 10 μg/ml) was prepared and listed in Figure 1. According to the OEPT, the content of tannin extracts was estimated to be 81%.

### Antibacterial activity

The micro-dilution method was used to study the inhibitory and bactericidal effects of tannin extracts against the pathogens. Tannin extracts showed inhibitory activity against gram- negative bacteria *Klebsiella pneumonia *(MIC value 0.3125 mg/ml and MBC value 0.625 mg/ml) and gram- positive bacteria *Staphylococcus aureus *(MIC value 0.3125 mg/ml and MBC value 1.25 mg/ml).

**Figure 2:**
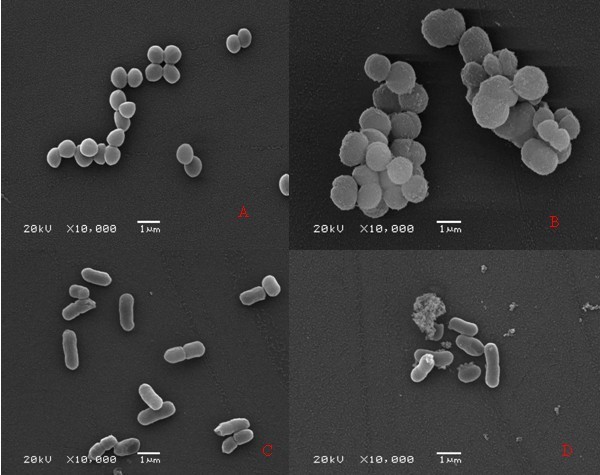
**Morphology of *Staphylococcus aureus *and *Klebsiella Pneumonia *under scanning electron microscope**. A the normal morphology of *Staphylococcus aureus*; B the morphology of *Staphylococcus aureus *on 24 h; C the normal morphology of *Klebsiella Pneumonia*; D the morphology of *Klebsiella Pneumonia *on 24 h

After treating the tannin extract, we observed that the *Staphylococcus aureus *and *Klebsiella pneumonia *had morphologic changes under electron microscope. The changes were obviously compared with the bacteria of the control group. A normal bacterium shows smooth surface, plump appearance, and good refraction. At 24 hours after treatment, majority of the bacteria of group II became obviously shrank, dry, and distorted. Some bacteria became obviously depressed, vesicular, or irregular. The changes showed that tannin extracts of a MIC concentration could obviously destroy those two kinds of bacteria. Results have been shown in Figure 2.

### Wound contraction

The areas of the original wounds of group I, II, and III were 1.81 ± 0.22 cm^2^, 1.80 ± 0.11 cm^2^, and 1.80 ± 0.19 cm^2^, respectively. As far as percentage of wound contraction was concerned, no significant difference was found among the groups on days 1, 3, 14, and 21 after wound creation. On days 7 and 10, percentage of wound contraction of either group II or III was higher than that of group I with significant difference (P < 0.05). All wounds were healed prior to day 21 (Additional file 1, Table S1).

### Histological examination of excised tissues

**Figure 3:**
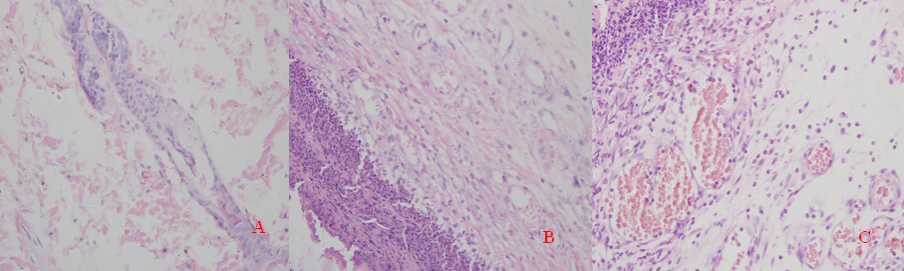
**Histological compare in the character of wound healing on day 3**. (100 × magnification). A: groupⅠ; B: groupⅡ; C: groupⅢ

On the first day after treatment, re-epithelialization, granulation, and collagen deposition were similarly developed in three groups. The wound showed migrating reepithelialization, abundant fibrinous exudation, few vessels, and trace collagen. At the third day, the wound of group II illustrated an excessive proliferation of granulation tissue. The wound of group III showed proliferation of newly formed micro-vessels and accumulation of inflammatory cells and fibroblast (Figure 3). At the seventh and tenth days, the wounds of all three groups displayed a continuous epithelial line that covered the whole wound bed. Wound granulation of group II and III was matured. The wound showed capillary vertically orientation, robust fusiform fibrocytes, and moderate well-arranged collagen. The granulation tissue of the wound of group I was not matured. The tissue had poorly organized capillary, multiple fibroblast, and slight collagen formation. Except for reepithelialization value, the mean values of parameter related to wound healing of group II and III were higher than that of group I (P < 0.05). On the fourteenth day, wound healing was observed in almost all the wounds. On the twentieth day, healing of wounds was observed in all wounds. In terms of histological changes, we found no significant difference among the three groups (Additional file 2, Table S2).

### Immunohistochemical examination of VEGFA expression

**Figure 4:**
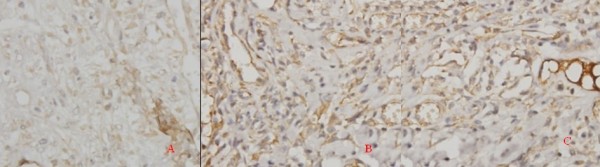
**Immunohistochemical examination of VEGFA expression on day 3**. (400 × magnification). A: groupⅠ; B: groupⅡ; C: group Ⅲ

On the first day, we found no significant difference among the three groups, in terms of the wound area, DM, and IOD of VEGFA expression. On the third day, the above parameters of group II and III were higher than those of group I, (P < 0.05). On the third day, in all groups, we observed the VEGFA expression at the climax along the curve of the whole healing process (Figure 4). The VEGFA expression decreased on the seventh and tenth days. In addition, we found no significant difference in VEGFA expression at other time points (Additional file 3, Table S3).

### VEGFA mRNA expression determined by RT-PCR

**Figure 5:**
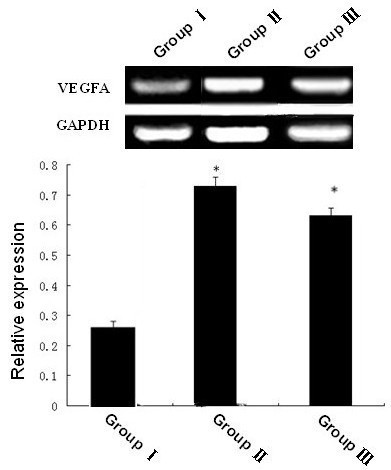
**VEGFA mRNA expression on day 3**. (*P < 0.05 as compared with groupⅠ)

On the third day, VEGFA mRNA expression of the wound was observed among all three groups after determining it through the RT-PCR technique (Figure 5). The expression of both group II and group III was higher than that of group I (P < 0.05). At other time points, no significant difference was reported in the expression among the three groups.

## Discussion

Botanical drugs such as sanguisorbae, rhubarb, acaciae catechu can be effective in the treatment of a thermal burn. Earlier experiments have attributed its effectiveness to tannin [21]. Tannins, polyphenolic compounds of various molecular weights, can be found abundantly in nature and have the ability to precipitate proteins [22]. With a molecular weight ranging from 500 and 4000 Da, these materials are soluble in water. They constitute a significant group of secondary ingredients of plants [23]. In the present study, based on evaluation of potentials of *Terminalia chebula Fructus Retz.*, we showed that tannin extracts has antimicrobial effects and application of the drug would improve wound healing.

When we extracted and purified tannin, we used water and alcohol for dissolution given the requirement of industrialized production as well as safety. It further determined the optimum extraction and purification technology (OEPT) of tannin extracts in order to ensure the high tannin content.

It is well-known that a wound is prone to bacterial infection, causing slow healing of wounds [24,25]. Recently, in addition to *Staphylococcus aureus*, drug-resistance bacteria, including *Klebsiella pneumonia *have been found in the wounds due to the abuse of antibiotics. Our study showed that tannin extracts have obviously inhibitory effect on both *Klebsiella pneumonia *and *Staphylococcus aureus*. With the aid of an electron microscope, we observed that the cell walls of the bacteria were destroyed. However, we need to further investigate if the anti-bacterial effect is correlated with the coagulating protoplasm of microbes or multiple enzymes.

In addition, tannin extracts from *Terminalia chebula Fructus Retz*. could up-regulate immune-histochemical, transcriptional, and translational levels of VEGFA expression, increasing the amount of newly formed capillaries at the inflammatory phase as well as the percentage of wound contraction at the granulation formation and scar remolding phases. In addition to promoting wound healing, when compared with erythromycin ointment or Vaseline, tannin extracts have a stronger angiogenic effect. We therefore believe that tannin extracts promote wound healing, probably through their associated powerful angiogenic property. Our results indicate that tannin extracts did not affect VEGFA expression at the later stages of healing process, thereby resulting in the acceleration of wound maturity. The reason may be related to neovascularity, an indicator of immature granulation tissue, diminishing gradually with the maturing of wound [15,20,26].

Recent studies showed that an infected wound usually had poor blood circulation [27,28]. Therefore, with effective blood concentration, an antibiotic could not reach the site of wound. In clinical practice, application of botanical drugs containing condensed tannin can obtain satisfied results. In addition to its direct anti-bacterial effect, it can also decrease the permeability of capillaries in the wound and alleviate tissue edema and exudation, resulting in rapid scab formation. As a result, it can effectively prevent the invasion of foreign microbes, avoiding enlargement and development of the infected wound. We need to further investigate if tannin extracts have a direct effect on an infected wound.

Although we preliminarily explored the mechanism of tannin extracts on the antibacterial effect promoting wound healing, the chemical properties, structure, physiological reaction, and pathological effects of tannin components have been unclear. Tannin may play a synergistic action with other chemical compositions. The action may interfere with other factors, thereby decreasing the effects of the botanical drug. We realize further study is necessary to reveal its mechanisms.

## Conclusions

Tannin extracts from immature fruits of *Terminalia chebula Fructus Retz*. can promote cutaneous wound healing in rats, which probably results from a powerful angiogenic and antibacterial activity.

## Competing interests

The authors declare that they have no competing interests.

## Authors' contributions

KL carried out the study and wrote the manuscript; HY supervised the work and the manuscript writing. BY and SSH prepared the plant extract. SYW and ZZ supervised the active experiment. YPD and HLZ contributed to the manuscript corrections and editing. All authors read and approved the final manuscript.

## Pre-publication history

The pre-publication history for this paper can be accessed here:

http://www.biomedcentral.com/1472-6882/11/86/prepub

## Supplementary Material

Additional file 1 **Table S1: The percent wound contraction at different time point**. The percent wound contraction was calculated on days 1, 3, 7, 10, 14 and 21 Click here for file

Additional file 2 **Table S2: Wound healing parameters of histological examination at different time point**. Wound healing parameters of histological examination was calculated on days 1, 3, 7, 10 and 14 Click here for file

Additional file 3 **Table S3: The expression of VEGFA at different time point**. The expression of VEGFA was observed on days 1, 3, 7, 10 and 14 Click here for file
