# Supplementary material for: Tannin extracts from immature fruits of Terminalia chebula Fructus Retz. promote cutaneous wound healing in rats
Source: BMC Complement Altern Med. 2011 Oct 7;11:86. doi: 10.1186/1472-6882-11-86 (PMC3198757; doi:10.1186/1472-6882-11-86)
Supplement: Additional file 1 — Table S1: The percent wound contraction at different time point. The percent wound contraction was calculated on days 1, 3, 7, 10, 14 and 21 [file 1472-6882-11-86-S1.DOC]

##

| Group | Wounds | Original wound area(cm^2^) | Percent wound contract (%) | | | | | |
| --- | --- | --- | --- | --- | --- | --- | --- | --- |
|  |  |  | Day 1 | Day 3 | Day 7 | Day 10 | Day 14 | Day21 |
| group Ⅰ | 6 | 1.81±0.22 | 7.11±0.21 | 18.90±1.75 | 51.18±1.67 | 70.23±1.55 | 96.12±1.77 | 100 |
| group Ⅱ | 6 | 1.80±011 | 7.11±0.14 | 19.22±1.83 | 65.32±1.98 ^*^ | 86.17±1.35 ^*^ | 100 | 100 |
| group Ⅲ | 6 | 1.80±0.19 | 7.13±0.18 | 19.31±1.91 | 66.14±1.85 | 88.21±2.17 | 100 | 100 |

##

Values are mean ± S.D. of six wounds in each group.

*P<0.05 as compared to groupⅠ.
