# Supplementary material for: Tannin extracts from immature fruits of Terminalia chebula Fructus Retz. promote cutaneous wound healing in rats
Source: BMC Complement Altern Med. 2011 Oct 7;11:86. doi: 10.1186/1472-6882-11-86 (PMC3198757; doi:10.1186/1472-6882-11-86)
Supplement: Additional file 2 — Table S2: Wound healing parameters of histological examination at different time point. Wound healing parameters of histological examination was calculated on days 1, 3, 7, 10 and 14 [file 1472-6882-11-86-S2.DOC]

| Group | Day 1 | | |  | Day 3 | | |  | Day 7 | | |  | Day 10 | | |  | Day 14 | | |
| --- | --- | --- | --- | --- | --- | --- | --- | --- | --- | --- | --- | --- | --- | --- | --- | --- | --- | --- | --- |
|  | Re-epithelialization | granulation | collagen |  | Re-epithelialization | granulation | collagen |  | Re-epithelialization | granulation | collagen |  | Re-epithelialization | granulation | collagen |  | Re-epithelialization | granulation | collegen |
| group Ⅰ | 1.21 | 1.51 | 0.77 |  | 2.55 | 2.38 | 2.58 |  | 3.04 | 2.75 | 2.81 |  | 3.48 | 3.15 | 3.29 |  | 3.89 | 3.94 | 3.91 |
| group Ⅱ | 1.20 | 1.50 | 0.80 |  | 2.60 | 2.58 ^*^ | 2.70 ^*^ |  | 3.07 | 3.68^*^ | 3.66 ^*^ |  | 3.54 | 3.91 ^*^ | 3.91 ^*^ |  | 3.91 | 3.98 | 4 |
| group Ⅲ | 1.20 | 1.49 | 0.78 |  | 2.60 | 2.99^*^ | 2.88 ^*^ |  | 3.06 | 3.66^*^ | 3.70 ^*^ |  | 3.57 | 3.95 ^*^ | 3.89 ^*^ |  | 3.95 | 4 | 4 |

Values are mean ± S.D. of six wounds in each group.

*P<0.05 as compared to group Ⅰ.
