# Supplementary material for: Tannin extracts from immature fruits of Terminalia chebula Fructus Retz. promote cutaneous wound healing in rats
Source: BMC Complement Altern Med. 2011 Oct 7;11:86. doi: 10.1186/1472-6882-11-86 (PMC3198757; doi:10.1186/1472-6882-11-86)
Supplement: Additional file 3 — Table S3: The expression of VEGFA at different time point. The expression of VEGFA was observed on days 1, 3, 7, 10 and 14 [file 1472-6882-11-86-S3.DOC]

| Group | Day 1 | | |  | Day 3 | | |  | Day 7 | | |  | Day 10 | | |  | Day 14 | | |
| --- | --- | --- | --- | --- | --- | --- | --- | --- | --- | --- | --- | --- | --- | --- | --- | --- | --- | --- | --- |
|  | Area(μm2) | Density mean | IOD |  | Area(μm2) | Density mean | IOD |  | Area(μm2) | Density mean | IOD |  | Area(μm2) | Density mean | IOD |  | Area(μm2) | Density mean | IOD |
| group Ⅰ | 72.32±4.13 | 0.30±0.09 | 19.11±9.41 |  | 89.14±12.35 | 0.33±0.33 | 28.13±5.11 |  | 112.35±8.33 | 0.34±0.08 | 37.12±6.12 |  | 116.71±1.84 | 0.38±0. 15 | 30.59±6.33 |  | 9.68±2.02 | 0.32±0.28 | 25.41±3.29 |
| group Ⅱ | 71.18±5.23 | 0.31±0.13 | 19.03±5.23 |  | 132.31±15.11* | 0.38±0.35 | 71.35±2.89* |  | 158.85±12.43 | 0.35±0.01 | 38.322±7.23 |  | 118.24±3.17 | 0.37±0.41 | 30.12±5.11 |  | 9.50±2.11 | 0.33±0.22 | 26.21±0.61 |
| group Ⅲ | 71.25±3.35 | 0.31±0.25 | 17.55±5.44 |  | 134.51±9.18* | 0.37±0.11 | 63.11±3.87* |  | 160.42±7.32 | 0.35±0.31 | 39.29±11.2 |  | 115.56±10.40 | 0.38±0.21 | 31.33±4.31 |  | 9.32±2.62 | 0.33±0.41 | 25.78±4.12 |

Values are mean ± S.D. of six wounds in each group.

*P<0.05 as compared to group Ⅰ
